# Co-evolution techniques are reshaping the way we do structural bioinformatics

**DOI:** 10.12688/f1000research.11543.1

**Published:** 2017-07-25

**Authors:** Saulo de Oliveira, Charlotte Deane

**Affiliations:** 1 Department of Statistics, University of Oxford, Oxford, UK

**Keywords:** Co-evolution techniques, Direct Coupling Analysis, structural bioinformatics

## Abstract

Co-evolution techniques were originally conceived to assist in protein structure prediction by inferring pairs of residues that share spatial proximity. However, the functional relationships that can be extrapolated from co-evolution have also proven to be useful in a wide array of structural bioinformatics applications. These techniques are a powerful way to extract structural and functional information in a sequence-rich world.

## Introduction

A large number of structural bioinformatics applications rely on extracting structural features from a protein’s sequence. This is traditionally done by performing multiple sequence alignments (MSAs) of homologues. MSAs have been used as input to predict features such as secondary structure, torsion and bond angles, solvent accessibility, disorder regions, and domain boundaries. The main limitation of most of these descriptors such as predicted secondary structure is that, although often highly accurate, they provide information only about a protein’s local conformation. For instance, they may tell us how a set of residues comprise an alpha-helix, but they do not provide any information as to how different alpha-helices are oriented with respect to one another. Techniques based on co-evolution go a step further by extracting non-local structural information from MSAs. These techniques are based on the notion that two residues which mutate in a correlated fashion, so that a mutation in one is often compensated by a mutation in the other, can be considered to be co-evolving. Co-evolution is interpreted as functional dependence, i.e. if two residues are co-evolving, there is a cost in fitness for mutating only one of these residues. Although these techniques were originally conceived and applied to protein structure prediction, they are now established tools with a diverse set of applications in structural bioinformatics.

Initial attempts at identifying co-evolving residues were implemented by calculating the correlation between columns in an MSA ^1– 6^. To quantify the precision of different methods, protein contacts (residues that share spatial proximity; usually C-βs less than 8 Å apart) were considered as true positives. These early attempts presented low precision and therefore limited usefulness. Methods based on calculating the Mutual Information (MI) between MSA columns were able to extend the applicability of these approaches ^7– 9^, but predictions were still not precise enough to be useful for most cases ^10^.

The exponential growth in the number of protein sequences combined with the application of existing statistical techniques that solved the inverse statistical problem to infer evolutionary couplings have allowed the development of methods with a precision range that has proved useful for many applications ^11– 14^. Direct Coupling Analysis (DCA) techniques are based on a generalised Ising model and, unlike MI and previous approaches, addressed the problem of transitivity by considering the correlation amongst all columns in the MSA as background to establish if two residues are co-evolving. Subsequent implementations based on similar ideas attempted to relax some of the assumptions of the original model and yielded progressively better results ^15– 18^. Although close in conception, these methods managed to produce a significant number of non-overlapping predicted correlations ^19^. Meta-predictors were then developed to combine the non-overlapping set of predictions to produce a consensus ^19– 21^, further improving the precision of co-evolution inference. A large-scale comparative study (~3,500 cases) has shown that the most precise of these methods, metaPSICOV ^22^, achieved a precision greater than 50% for its top L predictions, where L is the protein length, for over 68% of test cases ^23^. Other methods were developed with specific applicability, such as inferring co-evolving residues in membrane proteins ^24– 26^ or between β-sheets ^27^. The precision of predicted correlated mutations has continued to increase with improved methods using physicochemical information ^28^ and ultra-deep learning ^29^.

## Co-evolution and protein structure prediction

The implementation of DCA led to consistent and accurate *de novo* structure prediction for both soluble ^14, 15, 30^ and transmembrane proteins ^31, 32^ when sufficient sequence information is available. Recent results from the critical assessment of methods of protein structure prediction ^33^ have shown that in the presence of a sufficiently accurate number of predictions, topology prediction can be performed consistently and accurately. However, a few challenges remain regarding the identification and assignment of domain boundaries, longer proteins, and, most importantly, for cases where the number of available sequences is insufficient for accurate co-evolution inference. This latter problem is the main limitation; without enough diverse sequence information, accurate evolutionary coupling inference is currently impossible. When considering the results of the Critical Assessment of methods for protein Structure Prediction (CASP), a blind community-wide experiment that evaluates different prediction methods, protein structure prediction has been applied to a large number of cases where the target structure was unknown, providing reliable large-scale information about unknown folds ^34^.

## Understanding protein–protein interactions in light of co-evolution

Co-evolution analysis of paired sequences from interacting proteins has been shown to be effective in identifying pairs of residues involved in complex formation ^35^. A subsequent study has shown that when the number of paired sequences exceeds the average length of the proteins in the complex, most of the co-evolving residues are in contact at the protein–protein interface ^36^.

Co-evolution has also been shown to assist in protein–protein docking during the rounds 28–35 of the critical assessment of prediction of interactions. A potential based on co-evolution inference called InterEVScore was used in conjunction with ZDock, SOAP-PP, and Rosetta refinement to produce correct predictions for 10 out of 18 targets ^37, 38^. Co-evolution has also been used to identify protein–protein interactions and was shown to predict the only two experimentally known interactions of the trp operon ^39^. The main limitation that co-evolution techniques encounter when used to infer protein–protein interactions is that these methods require a large number of pairs of protein sequences of the same organism, which currently restricts its applicability to a small number of cases. Furthermore, pairing of same-organism sequences is particularly difficult in the presence of paralogues, and methods have been proposed to address this problem ^40, 41^.

## Co-evolving residues may be suggestive of multimerisation

The concept of using co-evolution to predict protein-protein interactions can be further extended to include protein multimerisation. This offsets the limitation of dependence on paired sequences. However, multimerisation prediction is more challenging than the identification of co-evolving residues in a protein–protein interaction interface, since it is necessary to discriminate between multimeric contacts and intra-monomer contacts.

Co-evolution techniques were used to correctly identify multimeric contacts for 18 dimeric complexes ^42^ and to validate a suggested dimeric interface between two Hsp70 molecules in the DnaK crystal ^43^. This success in multimeric prediction suggests that the existing quality assessment of co-evolution techniques may be underestimating their precision. This is because of the fact that pairs of residues interacting in the multimeric conformation would not necessarily share spatial proximity when considering the monomeric protein chain and thus would be incorrectly considered as false positives.

## Predicting domain boundaries by means of correlated mutations

Domain boundary identification is particularly useful for, but not restricted to, protein structure prediction, and it has been reported as one of the main challenges encountered in the free-modelling category of CASP ^44^. Protein contacts have been used for automatic domain boundary assignment and prediction by means of minimising the inter-domain contacts whilst maximising the number of intra-domain contacts ^45, 46^. However, these contact-based methods depend on an existing structure for the target sequence and are therefore not applicable when predicting new structures. This limitation, however, can potentially be overcome by using co-evolution inference to predict protein contacts. Correlated mutations output by MI led to the successful prediction of domain boundaries ^47^. A more precise co-evolution inference method has also been used for domain prediction. It was shown to produce better results for 368 targets compared to sequence-based methods and comparable results to homology-based methods ^48^.

## Identifying alternative conformations, allostery, and flexibility by means of co-evolution

Co-evolution provides a way of assessing the biological relevance of different conformations observed in coarse-grained structural-based models or molecular dynamics simulations. Co-evolving residues have been used to guide coarse-grained simulations either towards the native conformation or to explore conformational ensembles that are supported by evolution ^49^. They have also been used to identify distinct functional conformational states suggested to be observed between apo and holo conformations ^50– 53^. In another study, co-evolution was used to identify a framework for allostery for the MutS DNA mismatch repair protein ^54^ by means of Statistical Coupling Analysis (SCA). This approach differs from the traditional DCA, as it aims to construct a network of co-evolving residues as opposed to performing the correlation assessment on a pairwise level.

Identification of alternative conformations and allostery using experimental techniques is challenging, suggesting co-evolution techniques may be a powerful tool for exploring and targeting conformational dynamics. The success of co-evolution approaches suggests that co-evolving residues can be in contact only in a subset of a protein’s conformations. Once again, this highlights that the precision of co-evolution methods may be underestimated if they are tested against a single protein structure.

## Co-evolution can assist in experimental determination

Structural models produced *ab initio* can be used to assist in crystallographic protein structure determination, particularly when no other structural information is available. In these scenarios, *ab initio* models are used in molecular replacement protocols to solve the phasing problem. However, this is limited by the quality and reliability of the input models. Up until the advent of more precise co-evolution methods, *ab initio* protein structure prediction led to poor modelling results for a large number of cases, including longer and/or multi-domain proteins. Co-evolution has broadened the applicability of models produced in the absence of a template, leading to more consistent and reliable predictions. Models generated *ab initio* in conjunction with co-evolution constraints have been shown to improve the success of molecular replacement ^55, 56^. Co-evolution has also been used to characterise the order in which macromolecular complexes self-assemble, complementing existing experimental data for those complexes ^57^.

## Expanding the applicability of co-evolution via metagenomics

The precision of co-evolution methods is known to be dependent on the number of non-redundant sequences used in the MSA ^13– 15, 19, 30^. Insufficient sequence information constitutes the main limitation for co-evolution techniques. In the absence of a minimal number of non-redundant sequences, the inferred evolutionary couplings are unlikely to suit any of the purposes mentioned thus far. The usefulness of the predictions is therefore restricted to protein families for which a sufficient number of non-redundant sequences is available. It was previously reported that approximately 25% of the protein families on Pfam ^58^ would have a sufficient number of sequences for reliable co-evolution inference ^17^. Metagenomics data have been used as a source for additional sequences, thus expanding the applicability of co-evolution ^59^. Ovchinnikov and colleagues used metagenomics to increase the number of MSA sequences and subsequently to predict the protein structure of an additional 614 protein families, 140 with no members with known structure. Metagenomics provides a wealth of sequence information that is yet to be explored in other applications of co-evolution techniques, such as protein–protein interaction prediction and functional characterisation.

## Functional characterisation and fitness estimation

A common application in bioinformatics is to predict the effect of a particular mutation on a phenotype. Given that co-evolution aims to capture correlated mutations, it can be used to quantify how likely a mutation is to be compensated for by a second mutation in another residue. This, in turn, provides a means of estimating the fitness cost for a particular mutation considering its effect based on co-evolving residues. A recent method, EVmutation, uses co-evolution to quantify the effects of multiple mutations on the phenotype ^60^. Though the method can be generalised for any organism, it was tested for 34 cases to identify deleterious mutations in humans, showing comparable results to state-of-the-art supervised methods.

Maximum entropy models, which serve as a basis for several co-evolution methods, can also provide insights on the fitness landscape of a particular protein family ^61– 63^. These methods can estimate an energy for a target sequence that can be interpreted as the compatibility of this sequence to the fitness landscape of its family.

Another application of co-evolution-based fitness estimation relates to bioengineering. Co-evolution can be used to identify pairs of residues which, if mutated, can alter a protein’s stability and/or function. This is particularly important when selecting hotspots for enzyme engineering ^64^. As an example, co-evolving site mutagenesis was used to improve protein thermostability of alpha-amylase ^65^. There are also examples where mutations in co-evolving positions were shown to be de-stabilising ^64^. Evolutionary couplings can also highlight residue interactions that are not known either because a structure is unavailable or because such relationships are not evident from structural data (e.g. unresolved residues). This provides additional insights into protein folding, stability, and function that can be explored by synthetic biology/bioengineering.

## Conclusions

The advent of precise methods for the identification of co-evolving residues has led to progress in many areas of structural bioinformatics. The limited applicability of these methods, usually constrained by the amount of sequence information available, may be offset by metagenomics efforts and the exponential growth in sequence information. This paves the way for co-evolution to become as pivotal to bioinformatics analyses as sequence alignments themselves. The functional relationships that can be derived from these predictions provide a source of additional data that goes beyond the realm of structural prediction, translating an abundant source of information (sequence) into biological signal. Though still in their infancy, many of the alternative applications of co-evolution show great promise, and we can expect to see many advances and new techniques in these areas over the coming years.
